# Haglund Deformity and Plantar Calcaneal Spur in Insertional Achilles Tendinopathy: Prevalence, Coexistence, and Radiographic Characteristics

**DOI:** 10.1002/jfa2.70228

**Published:** 2026-10-08

**Authors:** İbrahim Halil Dadir

**Affiliations:** ^1^ Department of Orthopaedics and Traumatology Van Training and Research Hospital Van Türkiye

**Keywords:** haglund deformity, heel pain, insertional achilles tendinopathy, plantar calcaneal spur

## Abstract

**Background:**

Insertional Achilles tendinopathy (IAT) is a common cause of posterior heel pain and has been associated with several osseous abnormalities around the calcaneus. Although Haglund deformity and plantar calcaneal spur (PCS) have each been linked to IAT, their coexistence and radiographic characteristics have not been comprehensively evaluated. This study compared the prevalence of Haglund deformity and PCS between patients with IAT and controls, assessed their coexistence, and examined radiographic features associated with IAT.

**Methods:**

This retrospective case‐control study included 224 adults who underwent weight‐bearing lateral ankle radiography between January 2022 and January 2024, comprising 107 patients with clinically diagnosed IAT and 117 controls. The presence of Haglund deformity and PCS, PCS length, Fowler–Philip angle, Brink angle, and calcaneal pitch angle were evaluated. Multivariable logistic regression analysis was performed to identify factors independently associated with IAT.

**Results:**

Haglund deformity (65.4% vs. 15.4%, *p* < 0.001) and PCS (64.5% vs. 18.8%, *p* < 0.001) were significantly more prevalent in patients with IAT than in controls. Their coexistence was also more common in the IAT group (50.5% vs. 6.0%, *p* < 0.001), and PCS length was greater (*p* < 0.001). No significant differences were observed in Fowler–Philip angle, Brink angle, or calcaneal pitch angle. After multivariable adjustment, Haglund deformity (adjusted OR, 7.86; 95% CI, 3.75–16.48; *p* < 0.001), PCS (adjusted OR, 3.97; 95% CI, 1.94–8.13; *p* < 0.001), and higher body mass index (adjusted OR, 1.11; 95% CI, 1.05–1.18; *p* < 0.001) remained independently associated with IAT.

**Conclusions:**

Haglund deformity and PCS were independently associated with IAT and frequently coexisted in affected patients. These findings suggest that multiple structural abnormalities may occur concurrently at the calcaneal enthesis. Combined radiographic assessment of Haglund deformity and PCS may improve the evaluation of patients with IAT.

## 1 Introduction

Insertional Achilles tendinopathy (IAT) is a common overuse‐related disorder accounting for more than 20% of all Achilles tendon disorders and typically presents with posterior heel pain, erythema, swelling, and functional limitation [1]. Conservative management remains the first‐line treatment for IAT and generally includes activity modification, nonsteroidal anti‐inflammatory drugs, physical therapy, orthotic support, and temporary immobilization. Although these treatment modalities are effective for the majority of patients, a substantial proportion of patients continue to experience persistent symptoms and functional limitations despite prolonged conservative management [2].

IAT has a multifactorial etiology involving both intrinsic and extrinsic factors, including lower‐extremity biomechanical abnormalities, systemic conditions, and repetitive mechanical overload [3]. Among the intrinsic factors, anatomical abnormalities around the calcaneus have attracted increasing attention because of their potential to alter the mechanical environment at the Achilles tendon insertion. Haglund deformity, characterized by a posterosuperior prominence of the calcaneus, has been proposed as one of these anatomical factors; however, its relationship with IAT remains controversial despite growing evidence suggesting an association [4]. Similarly, plantar calcaneal spur (PCS) is a bony outgrowth of the calcaneal tuberosity that is associated with mechanical stress and repetitive traction forces. Although the relationship between PCS and Haglund deformity remains incompletely understood, recent radiographic studies have demonstrated a significant association between these two osseous abnormalities, suggesting that they may share common biomechanical stress pathways [5].

Although Haglund deformity and plantar calcaneal spur have each been associated with heel pain, few studies have simultaneously evaluated their prevalence, coexistence, and radiographic characteristics in patients with IAT.

Therefore, the present study aimed to compare the prevalence of Haglund deformity and PCS between patients with IAT and a control group, evaluate their coexistence, and characterize the radiographic features associated with IAT.

## 2 Methods

### 2.1 Study Design and Patient Selection

This retrospective case‐control study was conducted at the Department of Orthopedics and Traumatology, Van Training and Research Hospital, Türkiye. The study protocol was approved by the Non‐Interventional Clinical Research Ethics Committee of Van Training and Research Hospital (Approval No: GOKAEK/2026‐04/13) and was conducted in accordance with the ethical principles of the Declaration of Helsinki.

The institutional electronic medical record system was retrospectively reviewed to identify consecutive adult patients who underwent lateral ankle radiography between January 2022 and January 2024. A total of 356 patients were initially screened. Following the application of the eligibility criteria, 224 patients were included in the final analysis and allocated into two groups: an insertional Achilles tendinopathy (IAT) group (*n* = 107) and a control group (*n* = 117).

The IAT group comprised patients presenting with pain localized to the Achilles tendon insertion who received a clinical diagnosis of IAT based on compatible clinical history and physical examination findings, including localized tenderness at the Achilles tendon insertion and pain aggravated by physical activity. Lateral ankle radiographs were used to support the clinical diagnosis and evaluate associated osseous abnormalities. The control group comprised patients without pain localized to the Achilles tendon insertion who underwent lateral ankle radiography for clinical indications unrelated to Achilles tendon disorders, including suspected fracture, ankle sprain, or ankle pain unrelated to the Achilles tendon.

Patients aged 18 years or older with complete clinical records and adequate‐quality lateral ankle radiographs were eligible for inclusion. Only radiographs suitable for evaluation of calcaneal morphology and radiographic measurements were included in the analysis.

Exclusion criteria included incomplete clinical or radiographic records, inadequate‐quality lateral ankle radiographs precluding accurate radiographic measurements, and previous Achilles tendon or calcaneal surgery.

### 2.2 Radiographic Assessment

Standard weight‐bearing lateral ankle radiographs were retrieved from the institutional picture archiving and communication system (PACS). The presence of Haglund deformity and PCS was initially assessed by the primary observer. For inter‐observer reliability assessment, a second orthopedic surgeon independently evaluated the presence of Haglund deformity and PCS while blinded to the patients' case/control status and to the primary observer's assessments. For intra‐observer reliability assessment, the primary observer repeated the evaluation of Haglund deformity and PCS presence after a 1‐month interval while blinded to the results of the initial assessment. PCS length, Fowler–Philip angle, Brink angle, and calcaneal pitch angle were measured by the primary observer using the digital measurement tools integrated into the PACS software. All linear measurements were recorded in millimeters and angular measurements in degrees.

### 2.3 Definition of Radiographic Variables

Haglund deformity was assessed on weight‐bearing lateral ankle radiographs using the Heneghan–Pavlov parallel pitch lines (PPL) method [6]. The inferior PPL was drawn tangential to the anterior calcaneal tubercle and the medial calcaneal tuberosity. The superior PPL was drawn parallel to the inferior line and tangential to the posterior lip of the talar articular facet. Haglund deformity was classified as present (PPL‐positive) when the posterosuperior calcaneal prominence projected above the superior PPL and as absent (PPL‐negative) when the prominence touched or remained below this line (Figure 1). PCS was defined as a bony projection arising from the plantar aspect of the calcaneal tuberosity and was recorded as present or absent. In patients with a PCS, spur length was measured as the maximum distance from the plantar cortex of the calcaneus to the tip of the spur and was recorded in millimeters (mm). The Fowler–Philip angle was measured between an inferior line tangent to the inferior margin of the calcaneocuboid joint and the plantar calcaneal tuberosity and a superior line tangent to the posterior calcaneal prominence at the Achilles tendon insertion (Figure 2). The Brink angle was measured between a line extending from the superior aspect of the anterior calcaneal process to the posterosuperior aspect of the calcaneus and the calcaneal inclination axis (Figure 3). The calcaneal pitch angle was measured as the acute angle between a line along the inferior surface of the calcaneus and the plantar weight‐bearing axis, defined by a line extending from the plantar margin of the calcaneal tuberosity to the plantar surface of the fifth metatarsal head (Figure 4). All angular measurements were recorded in degrees (°).

**FIGURE 1:**
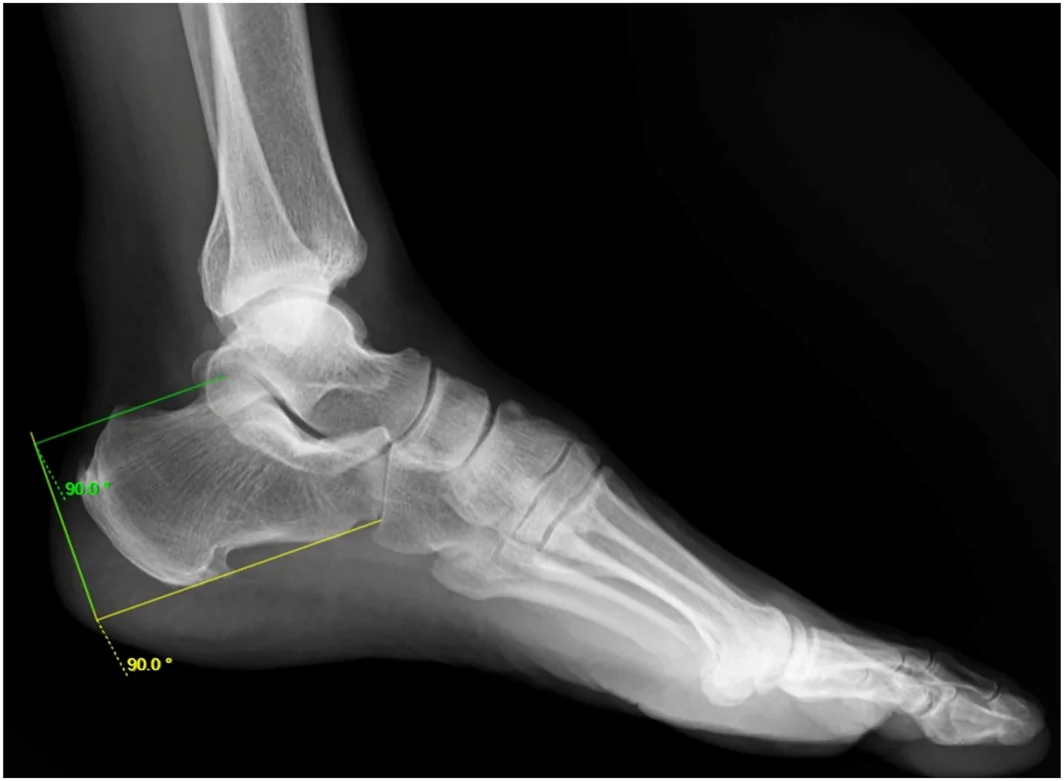
Assessment of Haglund deformity using the Heneghan–Pavlov parallel pitch lines on a weight‐bearing lateral ankle radiograph.

**FIGURE 2:**
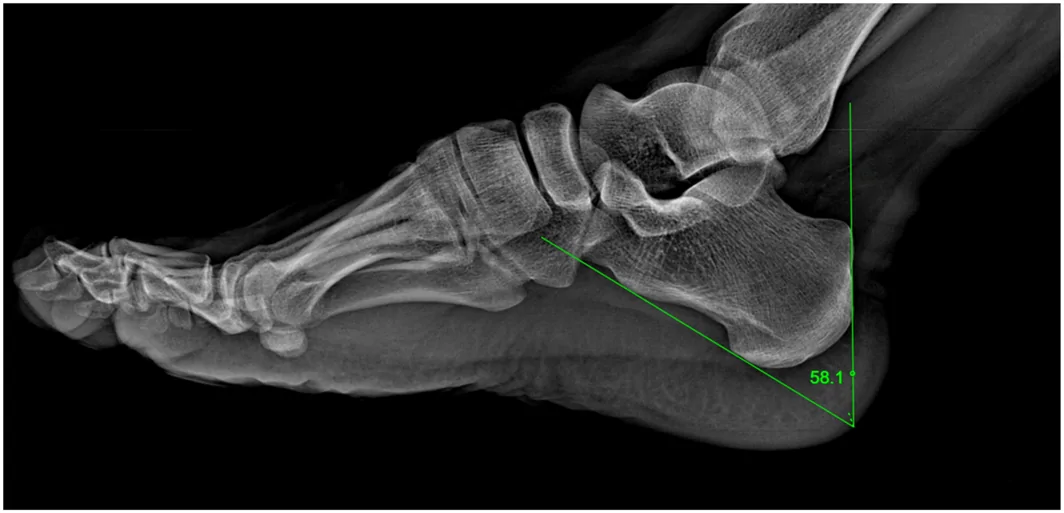
Measurement of the Fowler–Philip angle on a weight‐bearing lateral radiograph.

**FIGURE 3:**
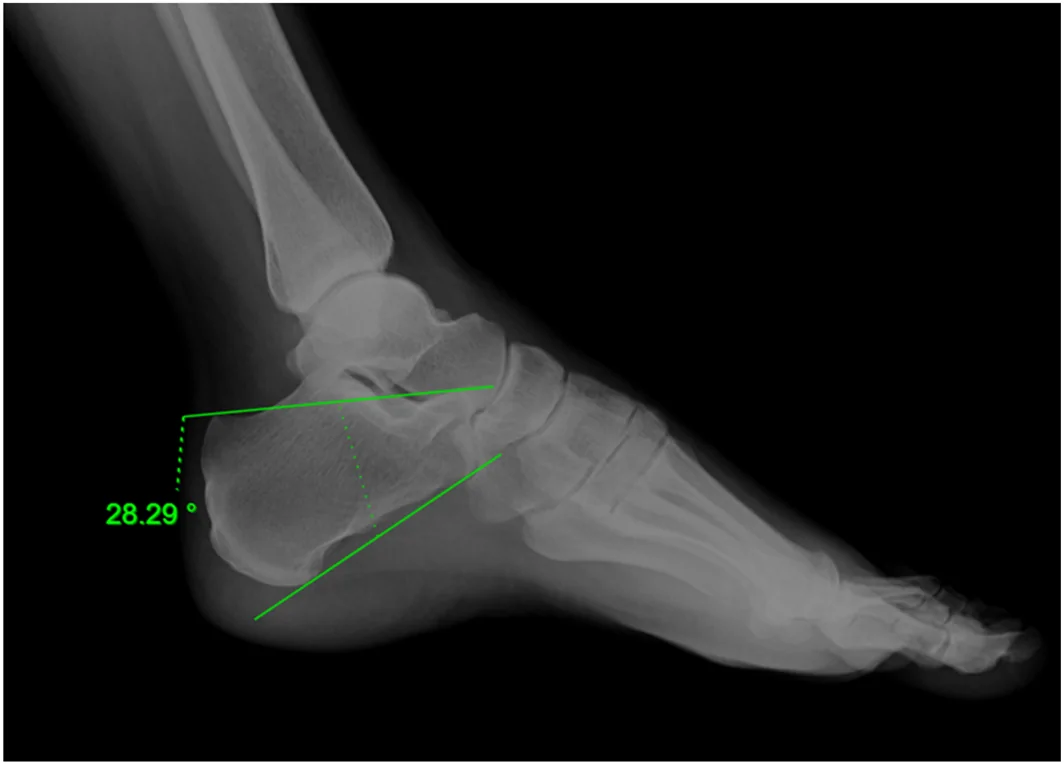
Measurement of the brink angle on a weight‐bearing lateral radiograph.

**FIGURE 4:**
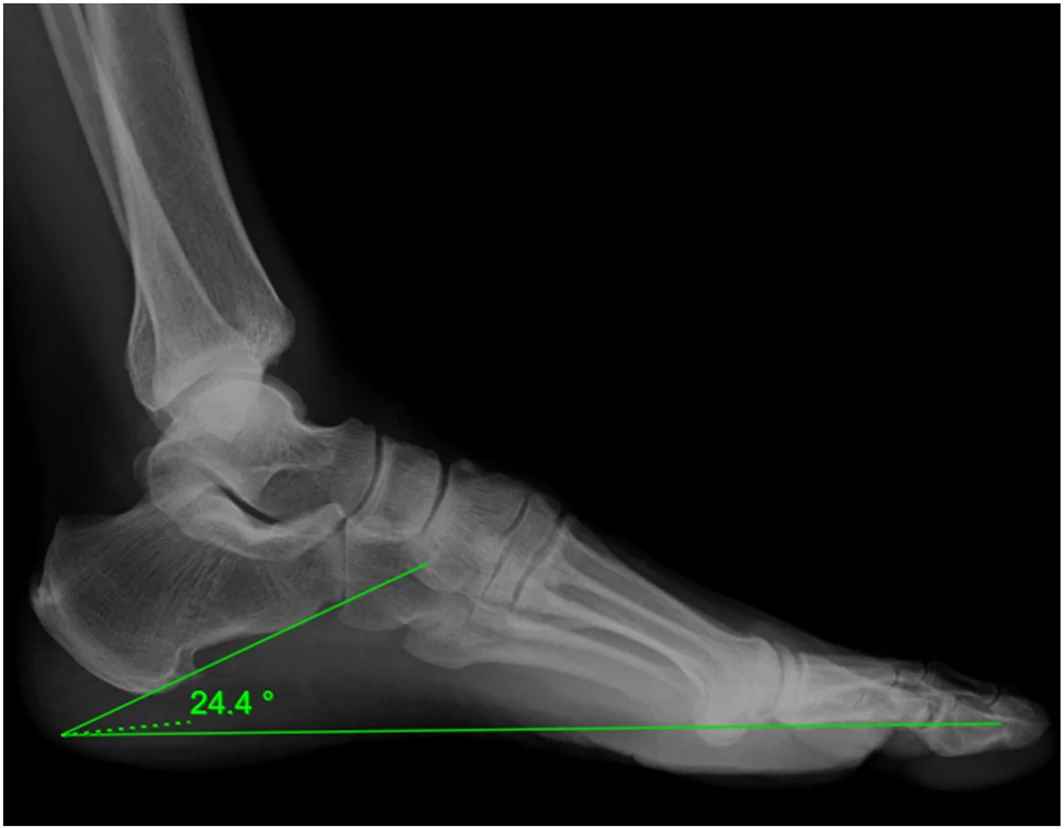
Measurement of the calcaneal pitch angle on a weight‐bearing lateral radiograph.

### 2.4 Clinical Variables

Demographic and clinical variables, including age, sex, affected side, body mass index (BMI), diabetes mellitus, and rheumatologic disease, were obtained from the electronic medical records. Rheumatologic diseases included rheumatoid arthritis, ankylosing spondylitis, gout, and psoriatic arthritis. Body mass index was calculated as weight in kilograms divided by height in meters squared (kg/m^2^).

### 2.5 Statistical Analysis

Statistical analyses were performed using IBM SPSS Statistics for Windows (version 30.0; IBM Corp., Armonk, NY, USA). The normality of continuous variables was assessed using the Shapiro–Wilk test. Continuous variables are presented as mean ± standard deviation (SD), whereas categorical variables are presented as frequencies and percentages. Comparisons between the IAT and control groups were performed using the independent‐samples Student's *t* test for normally distributed continuous variables and the Mann–Whitney *U* test for non‐normally distributed continuous variables. Categorical variables were compared using the Pearson chi‐square test or Fisher's exact test, as appropriate. For continuous radiographic variables, mean differences between groups were also reported with corresponding 95% confidence intervals (CIs). To identify factors associated with IAT, a multivariable logistic regression analysis was performed. Variables entered into the multivariable model included age, body mass index, diabetes mellitus, rheumatologic disease, Haglund deformity, and PCS. With 107 IAT cases and six predictors included in the primary multivariable model, the events‐per‐variable ratio was approximately 17.8. Adjusted odds ratios (ORs) with corresponding 95% confidence intervals (CIs) were calculated. As a sensitivity analysis, the multivariable logistic regression model was additionally adjusted for sex to account for the absence of age‐ and sex‐matching between the IAT and control groups. Multicollinearity among variables included in the multivariable logistic regression model was assessed using variance inflation factors (VIFs). Inter‐observer and intra‐observer agreement for the presence of Haglund deformity and PCS was assessed using Cohen's kappa coefficient (κ), with corresponding 95% confidence intervals (CIs). Percentage agreement was also calculated. A two‐sided *p* value < 0.05 was considered statistically significant.

## 3 Results

A total of 356 patients were initially screened for eligibility. After the application of the eligibility criteria, 224 patients were included in the final analysis, comprising 107 patients with IAT and 117 control subjects.

The baseline demographic and clinical characteristics of the study population are presented in Table 1. There were no significant differences between the IAT and control groups regarding age (42.0 ± 10.8 vs. 43.1 ± 12.8 years, *p* = 0.488), sex distribution (*p* = 0.666), side of involvement (*p* = 0.370), or the prevalence of diabetes mellitus (*p* = 0.652).

**TABLE 1:** Baseline demographic and clinical characteristics of patients with insertional achilles tendinopathy and the control group.

Variables	IAT (*n* = 107)	Control (*n* = 117)	*p*‐value
Age (years), mean ± SD	42.0 ± 10.8	43.1 ± 12.8	0.488
Sex, *n* (%)			0.666
Male	58 (54.2)	59 (50.4)	
Female	49 (45.8)	58 (49.6)	
Side, *n* (%)			0.370
Right	54 (50.5)	51 (43.6)	
Left	53 (49.5)	66 (56.4)	
BMI (kg/m^2^), mean ± SD	31.0 ± 7.1	27.4 ± 5.2	< 0.001
Diabetes mellitus, *n* (%)			0.652
Present	14 (13.1)	12 (10.3)	
Absent	93 (86.9)	105 (89.7)	
Rheumatologic disease, *n* (%)			0.040
Present	15 (14.0)	6 (5.1)	
Absent	92 (86.0)	111 (94.9)	

*Note:* Data are presented as mean ± standard deviation (SD) for continuous variables and as number (percentage) for categorical variables.

Abbreviations: BMI, body mass index; IAT, insertional Achilles tendinopathy; SD, standard deviation.

However, patients with IAT had a significantly higher body mass index than the control group (31.0 ± 7.1 vs. 27.4 ± 5.2 kg/m^2^, *p* < 0.001). In addition, rheumatologic disease was more frequently observed in the IAT group than in the control group (14.0% vs. 5.1%, *p* = 0.040).

Radiographic characteristics are summarized in Table 2. Haglund deformity was significantly more prevalent in patients with IAT than in controls (65.4% vs. 15.4%, *p* < 0.001). Similarly, PCS was observed more frequently in the IAT group (64.5% vs. 18.8%, *p* < 0.001). The coexistence of Haglund deformity and PCS was also markedly more common in patients with IAT than in controls (50.5% vs. 6.0%, *p* < 0.001). Furthermore, PCS length was significantly greater in the IAT group (6.15 ± 1.75 vs. 4.57 ± 1.10 mm, *p* < 0.001). In contrast, no significant differences were identified between the groups regarding Brink angle (*p* = 0.714), Fowler–Philip angle (*p* = 0.249), or calcaneal pitch angle (*p* = 0.497).

**TABLE 2:** Radiographic characteristics of patients with insertional achilles tendinopathy and the control group.

Variables	IAT (*n* = 107)	Control (*n* = 117)	Mean difference (95% CI)	*p*‐value
Haglund deformity, *n* (%)				< 0.001
Present	70 (65.4)	18 (15.4)		
Absent	37 (34.6)	99 (84.6)		
Plantar calcaneal spur, *n* (%)				< 0.001
Present	69 (64.5)	22 (18.8)		
Absent	38 (35.5)	95 (81.2)		
Coexistence of haglund deformity and plantar calcaneal spur, *n* (%)				< 0.001
Present	54 (50.5)	7 (6.0)		
Absent	53 (49.5)	110 (94.0)		
Plantar calcaneal spur length (mm), mean ± SD	6.15 ± 1.75	4.57 ± 1.10	1.58 (0.95–2.21)	< 0.001
Brink angle (°), mean ± SD	25.10 ± 4.14	25.28 ± 2.83	−0.18 (−1.12 to 0.77)	0.714
Fowler–Philip angle (°), mean ± SD	61.88 ± 10.59	60.35 ± 9.01	1.53 (−1.07–4.13)	0.249
Calcaneal pitch angle (°), mean ± SD	26.59 ± 4.93	26.20 ± 3.59	0.40 (−0.75–1.54)	0.497

*Note:* Data are presented as mean ± standard deviation (SD) for continuous variables and as number (percentage) for categorical variables. Mean differences were calculated as IAT group minus control group. PCS length was evaluated only in patients with a plantar calcaneal spur.

Abbreviations: IAT, insertional Achilles tendinopathy; PCS, plantar calcaneal spur; SD, standard deviation.

The results of the multivariable logistic regression analysis are presented in Table 3. After adjustment for age, body mass index, diabetes mellitus, rheumatologic disease, Haglund deformity, and PCS, both Haglund deformity (adjusted OR, 7.86; 95% CI, 3.75–16.48; *p* < 0.001) and PCS (adjusted OR, 3.97; 95% CI, 1.94–8.13; *p* < 0.001) remained significantly associated with IAT. Higher BMI was also independently associated with IAT (adjusted OR, 1.11; 95% CI, 1.05–1.18; *p* < 0.001). In contrast, age (*p* = 0.804), diabetes mellitus (*p* = 0.870), and rheumatologic disease (*p* = 0.059) were not significantly associated with IAT in the adjusted model. No evidence of problematic multicollinearity was identified in the multivariable model, with VIF values ranging from 1.02 to 1.33; specifically, the VIF values were 1.30 for Haglund deformity and 1.33 for PCS.

**TABLE 3:** Multivariable logistic regression analysis of factors independently associated with insertional achilles tendinopathy.

Variable	Adjusted OR	95% CI	*p*‐value
Haglund deformity	7.86	3.75–16.48	< 0.001
Plantar calcaneal spur	3.97	1.94–8.13	< 0.001
Age	1.00	0.97–1.04	0.804
Body mass index	1.11	1.05–1.18	< 0.001
Diabetes mellitus	0.90	0.26–3.08	0.870
Rheumatologic disease	3.21	0.96–10.76	0.059

*Note:* Adjusted odds ratios were estimated using a multivariable logistic regression model including age, body mass index, diabetes mellitus, rheumatologic disease, Haglund deformity, and plantar calcaneal spur.

Abbreviations: CI, confidence interval; IAT, insertional Achilles tendinopathy; OR, odds ratio.

In a sensitivity analysis additionally adjusting for sex, the results remained materially unchanged. Haglund deformity (adjusted OR, 8.00; 95% CI, 3.80–16.87; *p* < 0.001), PCS (adjusted OR, 3.93; 95% CI, 1.92–8.05; *p* < 0.001), and higher BMI (adjusted OR, 1.12; 95% CI, 1.05–1.18; *p* < 0.001) remained independently associated with IAT, whereas sex was not significantly associated with IAT (adjusted OR, 1.19; 95% CI, 0.59–2.37; *p* = 0.627).

Inter‐observer and intra‐observer agreement for the presence of Haglund deformity and PCS are presented in Table 4. For Haglund deformity, intra‐observer agreement was 97.3% (κ = 0.944; 95% CI, 0.899–0.988), and inter‐observer agreement was 96.0% (κ = 0.916; 95% CI, 0.862–0.970). For PCS presence, intra‐observer agreement was 95.5% (κ = 0.908; 95% CI, 0.852–0.964), and inter‐observer agreement was 86.6% (κ = 0.722; 95% CI, 0.630–0.815).

**TABLE 4:** Inter‐observer and intra‐observer agreement for the radiographic assessment of haglund deformity and plantar calcaneal spur.

Radiographic finding	Reliability	Agreement (%)	Cohen's κ	95% CI
Haglund deformity	Intra‐observer	97.3	0.944	0.899–0.988
Haglund deformity	Inter‐observer	96.0	0.916	0.862–0.970
Plantar calcaneal spur	Intra‐observer	95.5	0.908	0.852–0.964
Plantar calcaneal spur	Inter‐observer	86.6	0.722	0.630–0.815

*Note:* κ, Cohen's kappa coefficient; CI, confidence interval. Intra‐observer agreement was determined by comparing the primary observer's initial and repeat assessments. Inter‐observer agreement was determined by comparing the primary observer's initial assessment with the independent assessment of the second observer.

## 4 Discussion

In the present study, both Haglund deformity and PCS were significantly more prevalent in patients with IAT than in controls. In addition, the coexistence of these two osseous abnormalities was markedly more frequent in the IAT group, and both Haglund deformity and PCS remained independently associated with IAT after multivariable adjustment.

Haglund deformity is characterized by a posterosuperior bony prominence of the calcaneus that may increase mechanical irritation at the Achilles tendon insertion. This anatomical abnormality has frequently been implicated in the development of IAT and posterior heel pain [7]. Kiriluk et al. emphasized that Haglund deformity commonly coexists with IAT, highlighting the close anatomical relationship between posterosuperior calcaneal prominence and pathology at the Achilles tendon insertion [8]. Similarly, Poutoglidou et al., in a meta‐analysis, reported that Haglund deformity is frequently associated with IAT, further supporting the concept that posterosuperior calcaneal prominence represents a common radiographic finding in patients with insertional Achilles pathology [9]. Consistent with these observations, our findings further support the association between Haglund deformity and IAT, with a substantial effect size that persisted after multivariable adjustment (adjusted OR, 7.86). Although direct quantitative comparison with the studies by Kiriluk et al. and Poutoglidou et al. is limited because they did not report directly comparable odds ratios for this association [8, 9], differences in study populations, case selection, radiographic definitions, and analytical approaches may contribute to variation in the observed strength of association. Importantly, in the present study, Haglund deformity was assessed using the objective Heneghan–Pavlov parallel pitch lines method by observers blinded to case/control status, thereby reducing the potential for detection bias related to qualitative, non‐blinded assessment.

Compared with Haglund deformity, the relationship between PCS and Achilles tendinopathy has received relatively less attention. Vulcano et al. reported that plantar spurs frequently coexist with Achilles tendinopathy, with 41.9% of affected patients demonstrating a concomitant plantar spur, suggesting a significant association between these two conditions [10]. Similarly, Zhu et al. demonstrated that, among patients with IAT, PCS were significantly more common in those with posterior calcaneal spurs than in those without posterior spurs. The authors proposed that this coexistence may reflect the biomechanical continuity between the Achilles tendon and the plantar fascia, whereby increased tensile forces acting on the calcaneus may contribute to enthesophyte formation at both insertion sites [11]. In the present study, PCS was significantly more prevalent in patients with IAT than in controls and remained independently associated with IAT after multivariable adjustment. Furthermore, the coexistence of PCS and Haglund deformity was significantly more frequent in the IAT group, supporting the concept that multiple traction‐related osseous changes may develop concurrently around the calcaneal enthesis.

He et al. reported that a higher BMI was associated with an increased risk of Achilles tendinopathy [12]. Scott et al. demonstrated that patients with Achilles tendinopathy had significantly higher BMI than individuals without Achilles pathology [13]. Kozlovskaia et al. reported in a systematic review that increased BMI may represent an important risk factor for Achilles tendinopathy [14]. Our findings are consistent with the existing literature, demonstrating that patients with IAT had significantly higher BMI than controls and that BMI remained independently associated with IAT after multivariable adjustment.

Although rheumatologic disease was significantly associated with the outcome in univariate analysis, this association did not retain statistical significance after adjustment (*P* = 0.059). The relatively small number of patients with rheumatologic disease in both groups may have limited statistical power, and this finding should therefore be interpreted cautiously.

Kang et al. found no significant differences in Fowler–Philip angles between patients with IAT and controls, suggesting that conventional radiographic angular measurements may have limited value in distinguishing symptomatic patients [15]. Nakajima et al. found that the calcaneal pitch angle did not differ significantly between patients with IAT and control subjects [16]. Similarly, our study demonstrated no significant differences in Fowler–Philip angle, Brink angle, or calcaneal pitch angle between patients with IAT and controls.

Several quantitative radiographic parameters have been proposed for the assessment of Haglund morphology. However, traditional measurements such as the Fowler–Philip angle have demonstrated inconsistent discriminatory performance, and previous studies have reported limited sensitivity and specificity of conventional radiographic indices for distinguishing Haglund deformity [17, 18]. The Brink angle is a more recently introduced quantitative parameter that has shown promising discriminatory ability and good observer reliability on weight‐bearing radiographs [19]. Nevertheless, because different radiographic parameters and thresholds have been proposed, the Fowler–Philip and Brink angles were analyzed in the present study as continuous morphological variables rather than as diagnostic thresholds. Haglund deformity was instead classified using the Heneghan–Pavlov parallel pitch lines method to provide a single, objective, and reproducible criterion for determining the presence or absence of Haglund deformity.

Collectively, our findings suggest that IAT may be associated with multiple structural changes around the calcaneus rather than a single isolated osseous abnormality. Accordingly, evaluating Haglund deformity and plantar calcaneal spur together, rather than as separate radiographic findings, may contribute to a more comprehensive understanding of the structural changes associated with IAT and provide a more holistic radiographic assessment of these patients.

One of the main strengths of the present study is the simultaneous evaluation of both Haglund deformity and PCS in patients with IAT. While previous studies have generally focused on these radiographic abnormalities individually, our study evaluated their prevalence, coexistence, and independent associations with IAT within a single multivariable analytical model. In addition, the inclusion of a control group and the use of standardized weight‐bearing lateral radiographs enabled direct comparison of radiographic characteristics between patients with IAT and controls without symptoms localized to the Achilles tendon insertion. This approach provides a more comprehensive assessment of structural changes around the calcaneal enthesis and contributes to the existing literature regarding their potential clinical relevance.

This study has several limitations. First, its retrospective single‐center design may have introduced selection bias and may have limited the generalizability of the findings. Second, the diagnosis of IAT was based on clinical assessment without routine ultrasonographic or magnetic resonance imaging confirmation. Although this reflects routine clinical practice, the absence of advanced imaging may have influenced diagnostic accuracy in some patients. Third, the control group consisted of patients who underwent lateral ankle radiography for clinical indications unrelated to Achilles tendon disorders rather than a strictly asymptomatic population. Therefore, the control group may not fully represent the calcaneal morphology and BMI distribution of the general asymptomatic population, which may introduce selection bias and limit the generalizability of the findings. Finally, no a priori sample size calculation was performed because of the retrospective design of the study. However, the primary multivariable model included six predictors for 107 IAT cases, corresponding to an events‐per‐variable ratio of approximately 17.8. Future prospective multicenter studies with larger patient populations are warranted to validate and expand upon our findings.

## 5 Conclusion

Haglund deformity and PCS were significantly more prevalent in patients with IAT than in controls and remained independently associated with IAT after multivariable adjustment. The coexistence of these two osseous abnormalities was also markedly more common in patients with IAT, supporting the concept that multiple structural changes may occur concurrently around the calcaneal enthesis. These findings support the combined radiographic assessment of Haglund deformity and PCS as part of the evaluation of patients with IAT.

## Author Contributions

**İbrahim Halil Dadir:** conceptualization, methodology, data curation, formal analysis, investigation, visualization, writing – original draft, writing – review and editing.

## Funding

The author has nothing to report.

## Ethics Statement

This retrospective study was approved by the Non‐Interventional Clinical Research Ethics Committee of Van Training and Research Hospital (Approval No: GOKAEK/2026‐04/13). The study was conducted in accordance with the ethical principles of the Declaration of Helsinki.

## Consent

The requirement for informed consent was waived by the institutional ethics committee because of the retrospective design of the study and the use of anonymized patient data.

## Conflicts of Interest

The author declares no conflicts of interest.

## Permission to Reproduce Material From Other Sources

The author has nothing to report.

## Data Availability

The data that support the findings of this study are available from the corresponding author upon reasonable request.
